# Microstructures and Properties of Sn2.5Ag0.7Cu0.1RE Composite Solders Reinforced with Cu-Coated Graphene Nanosheets Synthesized by Pyrolysis

**DOI:** 10.3390/ma12020289

**Published:** 2019-01-17

**Authors:** Meng Zhang, Ke-ke Zhang, Fu-peng Huo, Hui-gai Wang, Yang Wang

**Affiliations:** 1 School of Materials Science and Engineering, Henan University of Science and Technology, Luoyang 471023, China; zmengaca@gmail.com (M.Z.); huofpyangm@163.com (F.-p.H.); hgwang_80@163.com (H.-g.W.); 2 Henan Key Laboratory of Non-ferrous Materials and Processing Technology, Henan University of Science and Technology, Luoyang 471023, China; 3 School of Materials Science and Engineering, University of Science and Technology Beijing, Beijing 100083, China; wangyang@xs.ustb.edu.cn

**Keywords:** pyrolysis, Cu-coated graphene nanosheets, Sn2.5Ag0.7Cu0.1RE composite solder, microstructures, properties

## Abstract

Composite solder is a promising route to improve the properties and reliability of Sn-based lead-free solder. In this study, Cu-coated graphene nanosheets (Cu-GNSs) were synthesized using pyrolysis. Cu-GNSs reinforced Sn2.5Ag0.7Cu0.1RE composite lead-free solders were prepared via powder metallurgy. The size, distribution, and adsorption type of Cu nanoparticles on the GNSs were studied. The relation of the Cu-GNSs content and microstructure to the physical, wettability, and mechanical properties of composite solders was discussed. The results show that Cu nanoparticles (with a mean size of 13 nm) present uniform distribution and effective chemisorptions on the GNS. Microstructural evolution of composite solders is dependent on the addition of Cu-GNSs. With increasing Cu-GNSs addition, β-Sn grains become finer and the eutectic phase proportion becomes larger, while the morphology of the eutectic phase transforms from dispersion to network-type. The improvement of the tensile strength of the composite solder can be attributed to grain refinement and load transfer. While the existence of Cu-GNSs can effectively improve the wettability and slightly change the melting point, it can also lead to the decline of elongation and electrical conductivity of the composite solder.

## 1. Introduction

Solder alloys act as the electrical and mechanical interconnection in the microelectronics package and serves an important role in the reliability and working life of electronic devices [1]. The modern design of high Input/Output density and three-dimensionality for microelectronics package technologies has inspired an urgent demand to develop novel lead-free solders of high-strength and toughness [2,3]. Microalloying and particle doping have been mainly utilized to improve the performance of lead-free solders. A series of commercial lead-free solders, such as Sn3.0Ag0.5Cu, have been developed using microalloying. Until recently, incorporating micro/nano particles into lead-free solders to prepare composite solders has come to the foreground as a hotspot. Especially carbon nanomaterials, including graphene and its derivative, carbon nanotubes (CNTs), etc. are used as reinforcements of composite solders due to their excellent thermal, electrical, and mechanical properties [4,5]. Nai et al. [6] were the first to dope CNTs into Sn-Ag-Cu-based solder and achieve an increment of wettability and microhardness. Subsequently, optimization of CNTs on the microstructure, tensile strength, and creep resistance was reported [7,8]. In view of the promising strengthening application of GNSs in the aluminum-based composite [9,10], Liu et al. [11] first prepared Sn-Ag-Cu-based composite solders reinforced with GNSs, and observed a resulting microstructural grain refinement and ultimate tensile strength improvement. The strengthening mechanisms of GNSs were attributed to grain refinement, dislocation strengthening, and load transfer [11,12]. However, the addition of GNSs has suffered from dilemmatic results in that the increment of the ultimate tensile strength and microhardness is at the cost of loss of ductility [9,11,13]. The loss of ductility was attributed to the poor bonding interface between GNSs and metallic solder matrix, because GNSs usually were pulled out of the matrix rather than serving as load-bearing bridges [11]. Authenticated spontaneous agglomeration of GNSs has also boosted the formation of microcracks [11,14]. Thus, metal nanoparticles have been selected to modify the surface of GNSs via electroless plating or other methods [15,16,17]. These metal nanoparticles not only form a transition layer with the matrix to facilitate the interfacial strength but also attain the relatively dispersive distribution of the GNSs [15]. For the system of Sn-Ag-Cu-based composite lead-free solder, the modification metals are preferable alloying elements such as Ni, Ag, and Cu, etc. which are compatible with the matrix [18]. Our previous studies proved that trace addition of Ni had positive effects on microstructure refinement and the discussed properties of solder and Ni-GNSs doped composite soldering joints [19,20]. Although the bonding strength between Cu and GNSs is less than the counterpart of Ni-GNSs, lattice defects of GNSs by high-energy milling give Cu nanoparticles the potential to form strong bonding with defected GNSs [21,22]. In addition, compared with Ag and Ni, Cu has the absolute advantage both in price and conductivity loss, which would further lower manufacturing costs of low-Ag Sn-based composite solder. However, there is no available literature about Cu coated GNSs strengthening low-Ag Sn-Ag-Cu-based composite solders.

In this paper, Cu coated GNSs (Cu-GNSs) and Sn2.5Ag0.7Cu0.1RE solder alloy were used as the reinforcement and matrix respectively, while a solid and green pyrolysis method (uniformly mixing and heating the high-energy milling treated GNSs and cooper acetate) was utilized to synthesize Cu-GNSs to avoid the loss of GNSs caused by iterative washing and filtering in the solution environment. Interfacial bonding types between Cu nanoparticles and GNSs were characterized by First-principles Calculations. Powder metallurgy was employed to prepare Sn2.5Ag0.7Cu0.1RE composite solders reinforced with Cu-GNSs. The microstructures and performances of the composite solders were investigated in detail.

## 2. Materials and Methods

### 2.1. Raw Materials

Sn2.5Ag0.5Cu0.1RE alloy powders (diameter 25–45 μm, TITD, Changsha, China) were used as matrix solder. The GNSs were produced via chemical reduction of graphene oxide (diameter 5–15 μm, thickness 5–25 nm, XFNANO, Nanjing, China) by hydrazine hydrate [23].

### 2.2. Synthesis of Cu-GNSs

Preparation of Cu-GNSs complex by pyrolysis included three steps of ball milling, mixing, and heating. Firstly, GNSs were milled for 3 h at 500 r/min in an XQM-0.4L planetary ball mill (MITR, Changsha, China), then the treated GNSs and copper acetate (Cu(CH_3_COO)_2_·H_2_O) powders were manually mixed, at a 10 mol% Cu loading ratio. Finally, mixed powders were heated in a tube furnace (Huayan, Shanghai, China) at 500 °C for 3 h under a nitrogen atmosphere and cooled to room temperature to obtain Cu-GNSs.

### 2.3. Preparation of Sn2.5Ag0.7Cu0.1RE/Cu-GNSs Composite Solders

Cu-GNSs were used as reinforcement to prepare a Sn2.5Ag0.7Cu0.1RE (SACR) composite lead-free solder reinforced with Cu-GNSs via the powder metallurgical route including powder mixing, compacting, sintering, and extrusion. First the pre-weighed Cu-GNSs powder was dispersed with a small amount of ethanol to form a uniform gray suspension by ultrasonic vibration (VGT-1730T, GT Sonic, Tianjin, China). Wet mixing was utilized to incorporate Cu-GNSs into Sn2.5Ag0.7Cu0.1RE alloy powders, and then the mixture was uniaxially compacted into a bulk at 160 MPa in a steel die and sintered at 210 °C under vacuum. After that, the solder bulk was extruded into a rod of diameter 10 mm. Five samples were made with addition of Cu-GNSs being 0 wt.%, 0.01 wt.%, 0.03 wt.%, 0.05 wt.%, and 0.10 wt.%, and designated as SACR, SACR/0.01Cu-GNS, SACR/0.03Cu-GNS, SACR/0.05Cu-GNS, and SACR/0.10Cu-GNS respectively.

### 2.4. Microstructure and Property Characterization of Cu-GNSs Complex

X-ray diffraction (XRD, Bruker D8-Advance, Billerica, MA, USA) with CuKα radiation was carried out to identify phases components of Cu-GNSs complex synthesized by pyrolysis, and the morphology of Cu-GNSs was characterized by a transmission electron microscope (TEM, JEOL, JEM-2100, Tokyo, Japan) and a high-resolution transmission electron microscope (HRTEM). In the planar hexagonal honeycomb structure of graphene, C atom lattice (top position) and the midpoints of the C–C covalent bonds (bridge position) are highly symmetric sites. Some metal atoms including Cu tend to adsorb at these two positions to form a stable adsorption system in the actual adsorption process [24,25]. Therefore, the DMol3 package of Material Studio software (BIOVIA, San Diego, CA, USA) based on the First-principles method [26] was used to calculate the adsorption energy and the height of the top/bridge position Cu adsorption on the intrinsic graphene (IG, before ball milling) and vacancy-defected graphene (VG, after ball milling) to evaluate the effect of ball milling on the adsorption stability. The electron exchange–correlation interactions were described using Perdew, Burke, Ernzerhof (PBE) potential combined with Generalized Gradient Approximating (GGA) [24]. The double numerical plus polarization (DNP) basis set was selected with cut-off energy of 300 eV [27]. The integration of the Brillouin zone was sampled by 3 × 3 × 1 K-point. During geometry optimization, all atoms were fully relaxed until the total energy was converged within 1 × 10^−5^ Hartree, with the maximum force and the maximum displacement tolerance being set at 0.002 Hartree/Å and 0.005 Å, respectively. The adsorption energy was obtained by the equation Eadsorption = ECu + Egraphene − ECu/graphene.

### 2.5. Microstructure and Property Characterization of Sn2.5Ag0.7Cu0.1RE/Cu-GNSs Composite Solders

#### 2.5.1. Microstructural Characterization

The specimen was etched by a methanol solution containing 8 wt.% hydrochloric acid. The microstructure was observed by a scanning electron microscope (SEM, JEOL JSM-5610LV, Tokyo, Japan) with EDS (Energy-dispersive X-ray Spectroscopy). Average sizes of β-Sn grains were measured via Image Pro Plus software (Media Cybernetics, Rockville, MD, USA) by referring to Ref. [16]. 

#### 2.5.2. Physical Properties

The melting point of composite solder was determined by differential scanning calorimetry (DSC, Netzsch STA409PC, Selb, Germany). The total weight of the DSC sample was about 15 mg, which was heated to 260 °C at a heating rate of 10 °C/min. The electrical resistivity ρ of the composite solders was calculated via the formula ρ = (0.017241/electrical conductivity) × 100%. The electrical conductivity test was carried out via a portable Sigma 2008B1 conductivity tester (Sigma, Saint Louis, MO, USA). An as-sanded specimen with dimensions of φ 20 mm × 30 mm was used in the test. A total of five points on the end face were measured for each composite solder specimen.

#### 2.5.3. Wettability

The wettability test of composite solder was measured according to the Chinese National Standard GB28770-2012 [28] (Test methods for solders). as follows: (1) 0.2 ± 0.01 g solder bulk was placed on the polished Cu substrate of 40 mm × 40 mm × 2 mm. (2) The assembled test sample was heated to 270 °C in the box-type furnace. (3) The sample was cooled in the air until the solder bulk had completely spread on the Cu substrate. Referring to Ref. [19], the wetting angle and spreading area of composite solder on the Cu substrate were measured via AutoCAD software (Autodesk, San Rafael, CA, USA).

#### 2.5.4. Mechanical Properties

**Figure 1:**
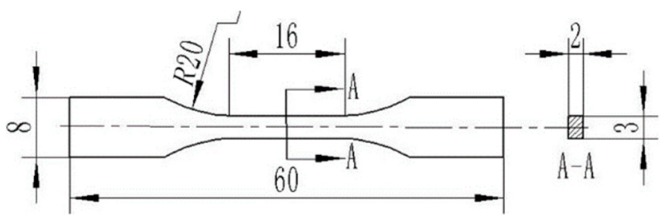
Tensile sample shape and dimension of composite solder (Unit: mm).

The specimen, as shown in Figure 1, was tensile tested on an AG-1250KN universal testing machine (SHIMADZU, Kyoto, Japan) at room temperature with a crosshead speed of 1 mm/min to obtain the tensile strength and elongation. The fractograph of the tensile specimen was observed and analyzed by means of SEM with EDS.

## 3. Results and Discussion

### 3.1. Characterization of Cu-GNSs

**Figure 2:**
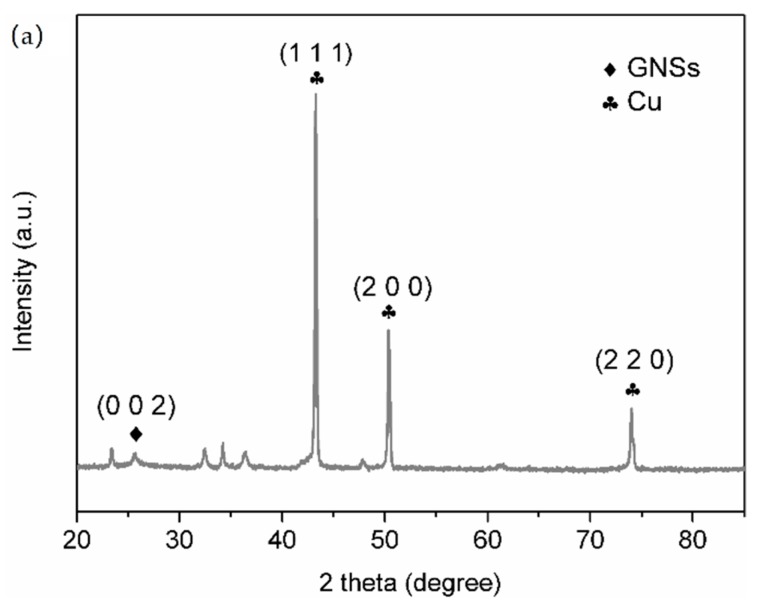

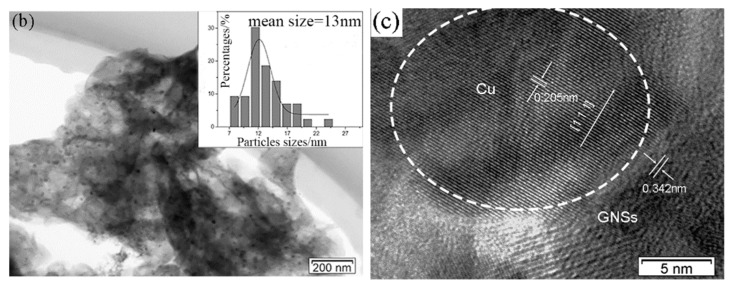
(**a**) X-ray diffraction pattern; (**b**) bright field transmission electron microscope image; (**c**) high-resolution transmission electron microscope HRTEM lattice fringe image of Cu-GNSs.

Figure 2 shows the XRD pattern and TEM morphology of Cu-GNSs. First of all, the diffraction peak at 26° corresponded to the (002) crystal plane of the GNSs (Figure 2a). According to the Bragg equation 2d·sinθ = n·λ (2θ = 26°), the interplanar spacing of Cu-GNSs was calculated to be 0.342 nm, which was the same as the theoretical spacing 0.34 nm of GNSs. This suggests that the pyrolysis did not change the GNSs layer structure and the layer structure of GNSs remained stable. In addition, the diffraction peaks at 43.29°, 50.43°, and 74.13° belong to the (111), (200), and (220) crystal planes of Cu (PDF#04-0836). Furthermore, no other phases were observed except the diffraction peaks of GNSs and simple Cu substance. The XRD pattern indicates that the copper acetate completely decomposed into the corresponding simple Cu substance after heating, which is consistent with the conclusion that the final decomposition product of Cu(CH_3_COO)_2_·H_2_O dehydration is Cu in the literature [29]. Additionally, TEM morphology of Cu-GNSs is shown in Figure 2b. The gray substrate is GNSs whose surface is uniformly covered with near-spherical black particles. The statistical results of the particle diameters showed that the diameters of near-spherical particles fell within the range of 7–25 nm and had a mean particle diameter of 13 nm. The HRTEM image of Cu-GNSs is shown in Figure 2c. The lattice fringe spacing of the central near-spherical particles is 0.205 nm, corresponding to the (111) crystal plane of Cu, indicating that the near-spherical particles are Cu nanoparticles. The marginal fringe spacing is 0.342 nm, which is consistent with the calculated GNSs layer spacing. These results ulteriorly show that the final product of pyrolysis is GNSs coated with Cu nanoparticles.

**Figure 3:**
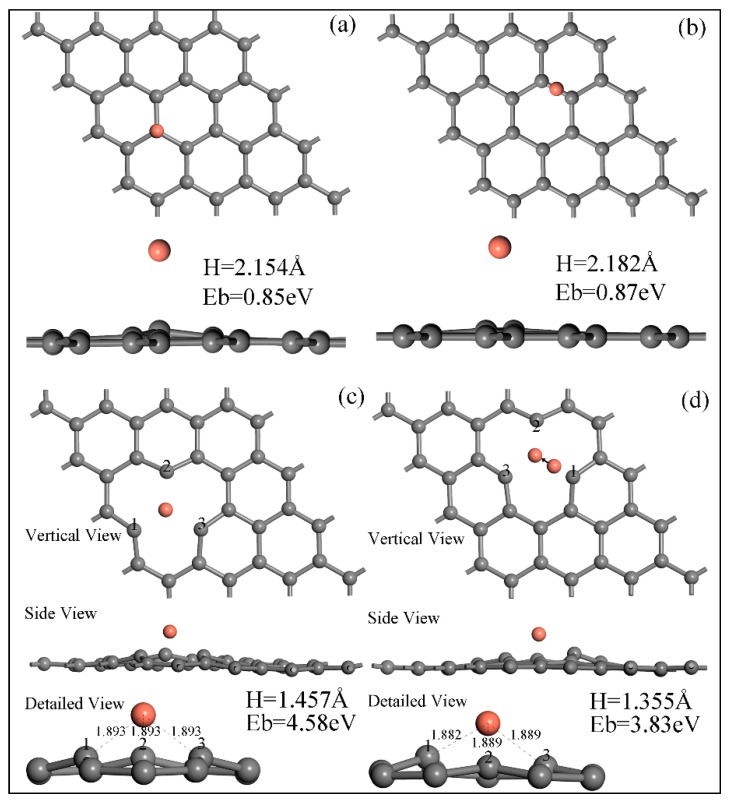
An atomic model of equilibrium adsorption systems. (**a**) Top Cu atom/intrinsic graphene; (**b**) bridge Cu atom/intrinsic graphene; (**c**) top Cu atom/vacancy-defected graphene; (**d**) bridge Cu atom/vacancy-defected graphene.

Based on the First-principle calculation, the equilibrium adsorption energy and adsorption height of the top/bridge position Cu atoms on the intrinsic/vacancy-defected graphene are shown in Figure 3. It can be seen that the adsorption energy of the top Cu atom/IG equilibrium system is 0.85 eV and the adsorption height is 2.154 Å (Figure 3a), which is lower than the counterpart (0.87 eV) of the bridge Cu atom/IG equilibrium system (Figure 3b). However, both of the adsorption heights are greater than the 1.92 Å of the covalent radius sum of Cu and C atoms, which indicates that the Cu/IG interface is a physisorption interface with weak bonding. In addition, during the high-energy ball milling, shear forces would delete certain carbon atoms in graphene to form VG [20]. The equilibrium adsorption energy and adsorption height of the top/bridge position Cu atoms on the VG are shown in Figure 3c,d, respectively. The side view in Figure 3c shows the VG had a sharp undulation, and the detailed view of Figure 3c shows obvious buckling of the nearest carbon atom marked 1–3. The buckling suggests that top Cu atom and nearest C atoms generated strong mutual attraction, forming three symmetrical Cu–C bonds. The adsorption height was reduced to 1.457 Å, and the adsorption energy increased to 4.58 eV. In this case, the adsorption type between Cu and VG belongs to chemisorption. This behavior agrees with the results that strong binding was formed in the Cu atoms adsorption doping VG system under similar adsorption parameters reported in the literature [22]. In the bridge Cu atom/VG equilibrium system, the Cu atom had a dramatic transfer from the bridge position to the top position, and finally stabilized near the top position, forming three asymmetric Cu–C bonds. The adsorption height was reduced to 1.355 Å, and the adsorption energy was raised to 3.83 eV (Figure 3d). Namely, the top position adsorption is the most stable adsorption structure for the Cu atom/VG system followed by the second bridge position adsorption. However, both of them belong to chemisorption. These results indicate that vacancy defects generated by high-energy ball milling increase the nucleation active sites of Cu nanoparticles on the surface of graphene, improve the adsorption capacity of graphene to Cu atoms, change the adsorption characteristics, and enhance the Cu/graphene interface strength.

In summary, the green and simple pyrolysis method can effectively realize the surface metallization of GNSs, and obtain Cu-GNSs reinforcement where uniformly distributed Cu nanoparticles form strong chemisorption with GNS.

### 3.2. Microstructure of Sn2.5Ag0.7Cu0.1RE/Cu-GNSs Composite Solder

**Figure 4:**
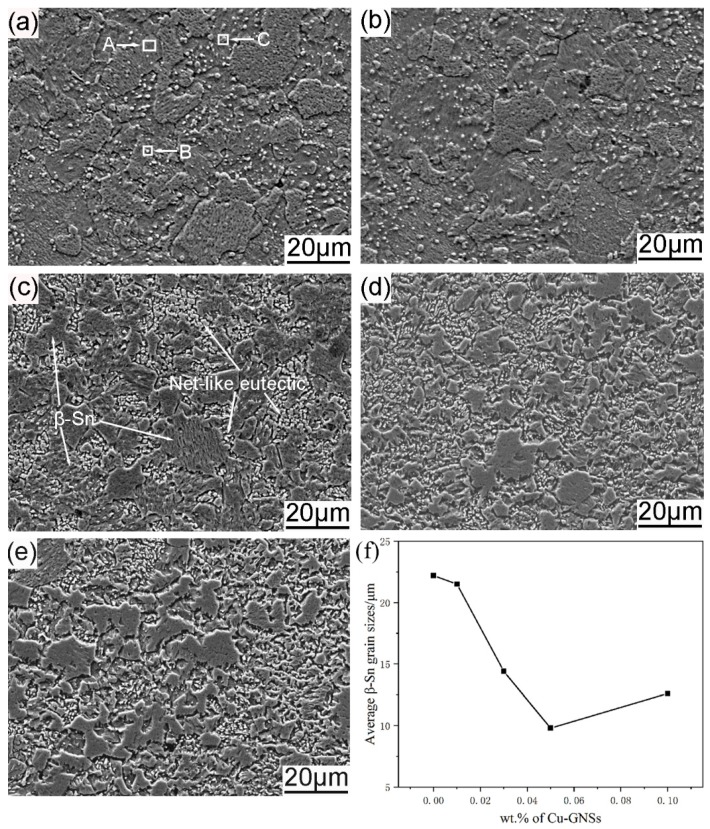
Microstructures of Sn2.5Ag0.7Cu0.1RE composite solder reinforced with (**a**) 0 wt.%; (**b**) 0.01 wt.%; (**c**) 0.03 wt.%; (**d**) 0.05 wt.%; (**e**) 0.10 wt.% Cu-GNSs; (**f**) average sizes of β-Sn grains.

The microstructure evolution of the SACR/Cu-GNSs composite solders are shown in Figure 4. The results of the corresponding EDS spectra analysis are shown in Table 1, taken from the regions A, B, and C in Figure 4a. Both Figure 4 and Table 1 show that the microstructure of SACR solder alloy consists of primary β-Sn phase and eutectic phases. The eutectic phases include β-Sn+Cu_6_Sn_5_, β-Sn+Ag_3_Sn, and β-Sn+Cu_6_Sn_5_+Ag_3_Sn while the microstructures of SACR/Cu-GNSs composite solders exhibited a decrease of average β-Sn grain sizes and increasing proportion of eutectic phases with Cu-GNSs addition. In addition, when the addition of Cu-GNSs is 0.03 wt.%, β-Sn grains in the microstructure of the composite solder are significantly refined and the morphologies of the eutectic phases change from dispersion to network as shown in Figure 4c. When the addition of Cu-GNSs is more than 0.05 wt.%, the microstructure of the composite solders presented no obvious change. 

**Figure 5:**
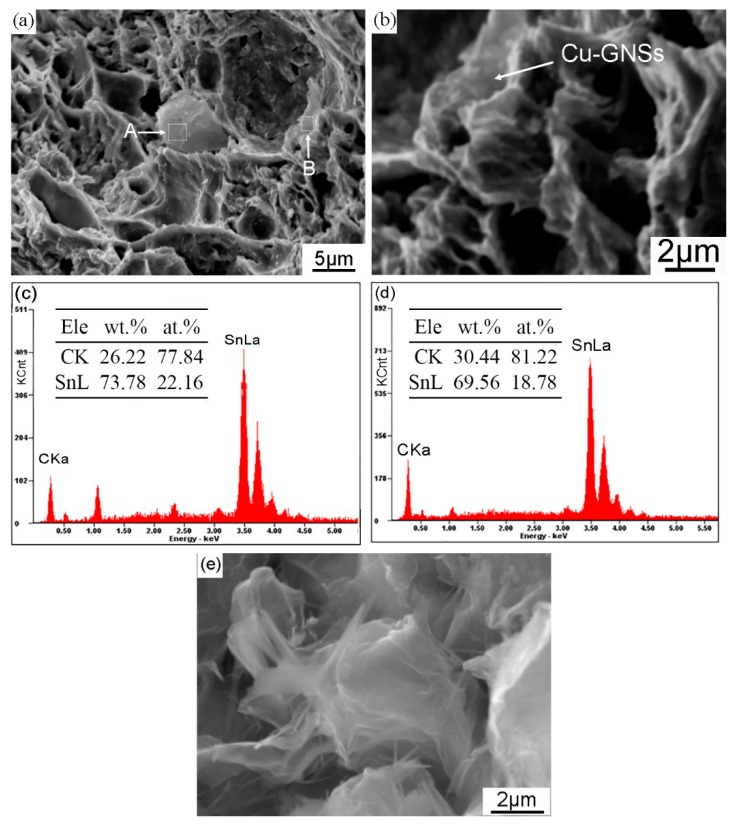
Scanning electron microscope micrographs of: (**a**) tensile fractograph of composite solder at 0.05 wt.% Cu-GNSs; (**b**) high magnification image of region B with Cu-GNSs clung to the edge of the dimple; (**c**,**d**) corresponding EDS spectra taken from the white rectangles marked as A and B in (**a**), respectively; (**e**) raw material of GNSs.

Huang et al. [12] reported that addition of GNSs resulted in a reduction of the grain size of β-Sn and an increase of eutectic phases in the microstructure of Sn3.0Ag0.5Cu composite solder. In view of the micro-scale plane of Cu-GNSs, the distribution of Cu-GNSs as observed by tensile fractograph of the composite solder is useful in understanding the detailed effect of Cu-GNSs on the microstructural evolution. Figure 5 shows the tensile fractograph of SACR/0.05Cu-GNS composite solder and the corresponding EDS spectra of selected regions. The tensile fractograph shows a typical dimple fracture as seen in Figure 5a. The energy spectra of sheets in the regions A and B, as shown in Figure 5c,d respectively, show that the carbon atomic fraction is over 70%. The 2D sizes of the sheets at the regions A and B are about 7 μm × 6.5 μm and 9 μm × 2 μm, which are similar to the geometric scale of as-reduced GNSs (5–15 μm) shown in Figure 5e. According to the carbon atomic fraction and 2D size, it can be deduced that the sheets in the regions A and B are Cu-GNSs. Further observation shows that Cu-GNSs in the region A cover the dimple surface around the pit. Figure 5b is a high magnification SEM image of Cu-GNSs in the region B which is close to the edge of the dimple. These results demonstrated that Cu-GNSs are randomly distributed in the SACR/0.05Cu-GNS composite solder matrix and the spontaneous aggregation of Cu-GNSs is restrained.

Combining the distribution of Cu-GNSs and the microstructural evolution of composite solders, one can see that large-size Cu-GNSs (region A) exist at the interface of the alloy powders to hinder the diffusion of matrix Sn atoms and the bonding of alloy powders, while small-size Cu-GNSs (region B) may be wrapped in metallurgical bonded alloy powders and exist at the grain boundary. Furthermore, these two forms of Cu-GNSs would inhibit the growth and coalescence of the β-Sn grains and refine the β-Sn grains.

### 3.3. Physical Properties of Sn2.5Ag0.7Cu0.1RE/Cu-GNSs Composite Solder

The DSC results of SACR/Cu-GNSs composite solder are shown in Table 2. The onset temperature and the peak temperature correspond to the solidus temperature and melting point respectively. As seen from Table 2, the melting point of the SACR solder alloy is 229.4 °C. When the addition of Cu-GNSs increases from 0.01 wt.% to 0.05 wt.%, the melting point of the composite solders exhibits a promising reduction, although the solidus temperature of SACR/Cu-GNSs rises slightly. Especially, the melting point of SACR/0.05Cu-GNS composite solder drops by 2.7 °C to 226.7 °C, which is close to the melting point (220 °C) of commercial Sn3.0Ag0.5Cu solder. For the composite solder system, the reduction of the melting point is attributed to the increased surface instability and surface free energy induced by reinforcement [30]. The above mentioned microstructural observation suggests that the addition of Cu-GNS results in a β-Sn grain refinement, which could explain the increase of surface free energy and the resulting decrease of melting point. However, other researchers declared no noticeable change in melting point after doping GNSs [15,16]. Because of the difference in observation sizes, the two viewpoints do not conflict each other. In a word, doping GNSs or CNTs provides an alternative route to tailor the thermal behaviors but does not limit the application of Sn-based composite solders.

The electrical resistivity of SACR/Cu-GNSs composite solder is shown in Table 2. It can be seen that the values of electrical resistivity of the matrix and composite solder fell within 12.2 ± 0.4 μΩ·cm with the increase of Cu-GNSs addition. This indicates that introduction of Cu-GNSs has no significant influence on the electrical resistivity of SACR/Cu-GNSs composite solder. A similar phenomenon was reported by previous researches [15,31]. The electrical resistivity of composite solder is composed of impurity, thermal and deformation resistivity affected by reinforcement, temperature, and lattice distortion [31]. In this paper, the inconspicuous change in electrical resistivity of SACR/Cu-GNSs composite solder may be attributed to the trace addition of Cu-GNSs. In addition, when the addition of Cu-GNSs is 0.03 wt.%, the electrical resistivity of SACR/0.03Cu-GNS composite solder is 12.1 μΩ·cm, corresponding to the best electrical conductivity, which is 11.7% lower than 13.7 Ω·cm of Sn3.0Ag0.5Cu as reported by Ref. [15].

### 3.4. Wettability of Sn2.5Ag0.7Cu0.1RE/Cu-GNSs Composite Solders

**Figure 6:**
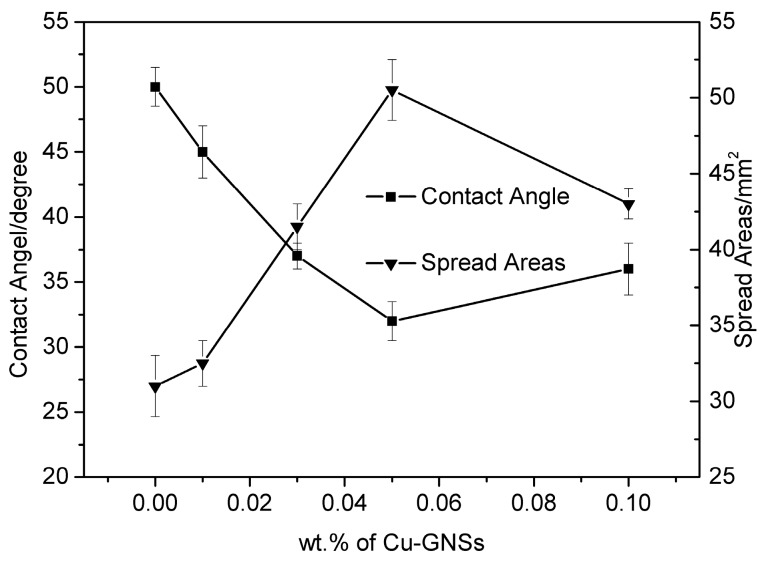
Contact angle and spreading areas of Sn2.5Ag0.7Cu0.1RE composite solders with various Cu-GNSs additions.

Wettability of SACR/Cu-GNSs composite solders on Cu substrate is characterized by the wetting angle and spreading area, as shown in Figure 6. In general, the spreading area of SACR/Cu-GNSs composite solders increases first and then decreases, whereas the corresponding wetting angle decreases first and then increases with Cu-GNSs addition. When the addition of Cu-GNSs is 0.05 wt.%, the spreading area of SACR/0.05Cu-GNS composite solder reaches to a 50.5 mm^2^ maximum which is higher than that of matrix solder. The spreading area of solder alloys is increased by 55% and the wetting angle shows a minimum of 32°, which is equivalent to the wetting performance of commercial Sn3.0Ag0.5Cu solder as reported by Ref. [15] and meets the microelectronic connection requirements for the wetting performance of novel lead-free solder alloys. Further addition of Cu-GNSs leads to a decrease of wettability of SACR/Cu-GNSs composite solder.

At present, there is no confirmative data to clarify the optimization mechanism of GNSs on the wettability of composite solders. Therefore, our paper attempts to submit an explanation. In the wetting process of graphene reinforced composite solder, GNSs tend to float to the melted solder/flux interface and even drift away with flux, due to the density difference between Cu-GNSs and melted solder [14]. However, interfacial GNSs disequilibrate the solid–liquid interface, and melted solder further spreads due to reduced surface tension [13]. Besides, because of the modification of Cu nanoparticles, the effective bonding formed between GNSs and melted solder enables Cu-GNSs to accumulate in the melted solder/flux interface, which could be beneficial for the sustained spreading of melted solder on the Cu substrate [15,17]. Thus a wettability improvement of SACR/Cu-GNSs composite solder as a function of Cu-GNSs addition was observed in this paper.

### 3.5. Mechanical Properties

**Figure 7:**
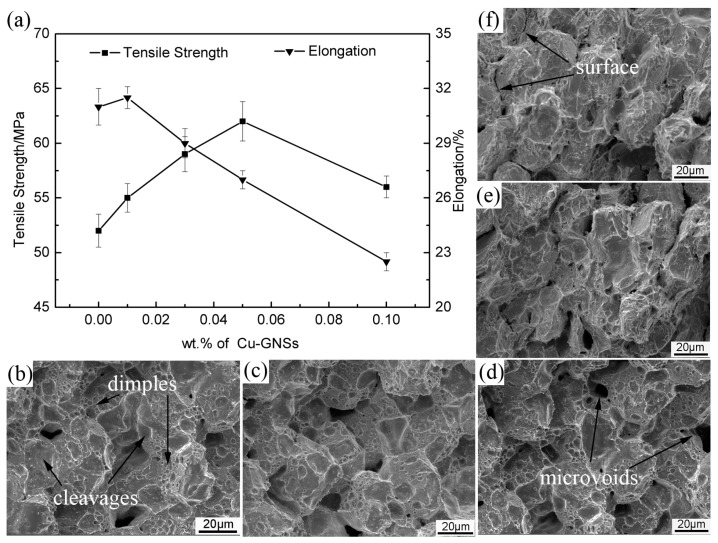
(**a**) Mechanical properties of Sn2.5Ag0.7Cu0.1RE/Cu-GNSs composite solders; and tensile fractographs corresponding to (**b**) 0 wt.%; (**c**) 0.01 wt.%; (**d**) 0.03 wt.%; (**e**) 0.05 wt.%; (**f**) 0.10 wt.% Cu-GNSs additions.

Figure 7 shows the mechanical properties and corresponding fractographs of SACR/Cu-GNSs composite solders. The tensile strength of SACR/Cu-GNS composite solders increases gradually and reaches a peak of 62 MPa at 0.05 wt.% Cu-GNSs addition, which is about 19% higher than that of matrix solder. However, elongation of SACR/Cu-GNS composite solders first increases slightly at 0.01 wt.% and then displays a significant decline from 0.01 wt.% (about 31.5%) to 0.10 wt.% (about 22%) of Cu-GNSs addition while the peaks of tensile strength and elongation have an undesired matching. When the addition of Cu-GNS is 0.03 wt.%, the best combination of mechanical properties is obtained with a tensile strength of 59 MPa and an elongation of 29%. This result is better than the counterpart as reported by Refs. [11,16,32].

The enhancement in tensile strength of SACR/Cu-GNSs composite solders can be explained by the connecting fractographs with microstructure. The fractographs of composite solders are shown in Figure 7b–f. With an increase of Cu-GNSs addition, grain refinement and an increase in area fraction of the eutectic phase can be seen in Figure 4, but no significant change in fractographs is observed (Figure 7b–d). This can lead to an enhancement of tensile strength [12,33]. On the other hand, Figure 5b shows some pulled out Cu-GNSs, demonstrating that the improvement of tensile strength is a benefit for load transfer [9]. However, the distinguishable primitive contour of alloy powders can gradually be observed in Figure 7e,f, and even a separated surface is visible. This corresponds to the decline of tensile strength. Besides, fractograph evolution indicates that over-load addition of Cu-GNSs has a negative effect on the sintering performance as evidenced by the appearance of a primitive contour and a separated surface.

As for elongation, a gradual decrease of dimple suggests a reduction of elongation. Furthermore, the above discussed primitive contour and separated surface can also make composite solder easier to form crack nucleation during deformation. Therefore, the SACR/Cu-GNSs composite solder presents falling elongation.

## 4. Conclusions

The following conclusions can be drawn from this study:

1. The dispersive and mean 13 nm Cu nanoparticles on the GNSs can be prepared by pyrolysis and the adsorption type transforms from physisorption to chemisorption after high-energy milling treatment of Cu-GNSs.
2. The trace addition of Cu-GNSs refined the β-Sn grains and increased the proportion of eutectic phases, while the morphology of the eutectic phases transforms from dispersion to network-type distribution.
3. The trace addition of Cu-GNSs could tailor the melting point of the composite solder.
4. At 0.03–0.05 wt.% addition of Cu-GNSs, the SACR/Cu-GNSs composite solder has the best-optimized performance combination in term of wettability and mechanical properties.
5. The enhancement of tensile strength can be attributed to grain refinement and load transfer.

## Figures and Tables

**Table 1:** Energy-dispersive X-ray Spectroscopy (EDS) analysis results (at.%) of region A, B, and C in Figure 4a.

Location	Sn	Ag	Cu
A	94.81	03.76	01.20
B	26.67	70.12	3.21
C	42.67	01.54	55.79

**Table 2:** Thermal properties and electrical resistivity of Sn2.5Ag0.7Cu0.1RE composite solder with various Cu-GNSs addition.

Solders	Onset Temperature (°C)	Peak Temperature (°C)	Electrical Resistivity (μΩ·cm)
Sn3.0Ag0.5Cu	-	220	13.7 [15]
SACR	216.8	229.4	11.9 ± 0.1
SACR/0.01Cu-GNS	218.7	228.1	12.2 ± 0.2
SACR/0.03Cu-GNS	218.7	226.8	12.1 ± 0.3
SACR/0.05Cu-GNS	218.1	226.7	12.4 ± 0.2
SACR/0.10Cu-GNS	218.7	229.4	12.5 ± 0.1
